# Peptide-Cellulose Conjugates on Cotton-Based Materials Have Protease Sensor/Sequestrant Activity

**DOI:** 10.3390/s18072334

**Published:** 2018-07-18

**Authors:** J. Vincent Edwards, Krystal R. Fontenot, Falk Liebner, Brian D. Condon

**Affiliations:** 1 Southern Regional Research Center, USDA, New Orleans, LA 70124, USA; krystal.fontenot@ars.usda.gov (K.R.F.); brian.condon@ars.usda.gov (B.D.C.); 2 Division of Chemistry of Renewable Resources, University of Natural Resources and Life Sciences Vienna, Konrad-Lorenz-Straße 24, A-3430 Tulln an der Donau, Austria; falk.liebner@boku.ac.at

**Keywords:** chronic wound dressings, nanocellulose, aerogels, proteases, human neutrophil elastase

## Abstract

The growing incidence of chronic wounds in the world population has prompted increased interest in chronic wound dressings with protease-modulating activity and protease point of care sensors to treat and enable monitoring of elevated protease-based wound pathology. However, the overall design features needed for the combination of a chronic wound dressing that lowers protease activity along with protease detection capability as a single platform for semi-occlusive dressings has scarcely been addressed. The interface of dressing and sensor specific properties (porosity, permeability, moisture uptake properties, specific surface area, surface charge, and detection) relative to sensor bioactivity and protease sequestrant performance is explored here. Measurement of the material’s zeta potential demonstrated a correlation between negative charge and the ability of materials to bind positively charged Human Neutrophil Elastase. Peptide-cellulose conjugates as protease substrates prepared on a nanocellulosic aerogel were assessed for their compatibility with chronic wound dressing design. The porosity, wettability and absorption capacity of the nanocellulosic aerogel were consistent with values observed for semi-occlusive chronic wound dressing designs. The relationship of properties that effect dressing functionality and performance as well as impact sensor sensitivity are discussed in the context of the enzyme kinetics. The sensor sensitivity of the aerogel-based sensor is contrasted with current clinical studies on elastase. Taken together, comparative analysis of the influence of molecular features on the physical properties of three forms of cellulosic transducer surfaces provides a meaningful assessment of the interface compatibility of cellulose-based sensors and corresponding protease sequestrant materials for potential use in chronic wound sensor/dressing design platforms.

## 1. Introduction

### Chronic Wounds and Protease-Modulating Dressings

Chronic wounds are characterized based on defective extracellular matrix reorganization, an absence of re-epithelialization at the wound boundary, and prolonged inflammation [1,2,3]. During prolonged inflammation, which a non-healing wound becomes ‘stalled’ in, unusually high proteolytic activity [4] often occurs leading to degradation of growth factors and extracellular matrix tissue, which play a major role in poor cell proliferation and failure of the wound to close [5]. Although wound care products have increased in sophistication in an attempt to keep pace with wound healing science and provide better treatment options, chronic wounds are still a major clinical problem and it is estimated that there are over 40 million people who suffer from chronic wounds worldwide [6].

Over the last twenty years, the development of dressings with design features to selectively remove excessively harmful levels of proteases from chronic wounds has become an increasing priority in wound care [7,8,9,10]. Excessive levels of proteolytic activity are a result of the high ingestion of neutrophils that do not lower in concentration as with acute wounds when the inflammatory phase is completed. Neutrophils are rich in proteases. Moreover high levels of neutrophil proteases in the chronic wound as Human Neutrophil Elastase (HNE), Matrix Metalloproteases (MMPs) as well as other serine protease keep the wound stalled in the inflammatory phase. Treatment with wound dressings that lower excessive protease activity and promote normal growth factor release in the wound environment are part of recommended for clinical guidance as one of the key steps in a systematic molecular approach to reversing the pathophysiology of chronic wounds [11]. Both MMPs and HNE have been the target of protease modulating dressings that remove excessive amounts of proteases from the wound bed [12,13]. Thus, the removal of excessive proteolytic activity, which is damaging to growth factors and the extracellular matrix (ECM) in the chronic wound, enables completion of the wound healing cycle [14]. To address elevated protease levels in chronic wounds the concept of protease-modulating dressings was first introduced for the treatment of the harmful effects of excessive proteolytic activity in chronic wounds [15]. The aim of protease modulating dressings (also termed protease sequestrant and protease lowering dressings) is to remove harmful levels of proteases from the wound bed while not impacting normal protease levels required for healing.

In recent years, recommended guidance for the use of protease-modulating dressings in a strategy termed ‘test and treat’ has been initiated based on the coordinated use of point of care protease diagnostics with protease-modulating dressings [7]. The approach of identifying elevated protease levels with point of care sensors detection coupled to dressing treatment is being used clinically although it is in the early stages of being adopted and proven universally, and recent studies have demonstrated the importance of testing for the presence of specific protease activities that are clinically deemed as harmful [16]. Accordingly, point of care protease diagnostics is now being widely researched, and at least one product is currently available [17]. However, the coupling of protease detection with protease modulating dressings in a single design platform has received relatively less attention notwithstanding the clinical issues of how effectively a sensor/dressing platform might be utilized or in what way it might be employed e.g., as a dressing ‘time to change’, in situ diagnostic of ELP, monitor of sustained release of protease inhibitor in the wound.

Accordingly the subject of this paper is to extend the spatiotemporal structure/function study presented previously [18] by examining the capacity of peptide immobilized cellulose analogs to detect proteases with sensor’s interfaced in situ with protease sequestrant material designed to work in concert with the sensor to lower elevated protease levels in the chronic wound. Previously we contrasted the molecular properties of a cotton-based nanocellulose aerogel (NA) material as a protease sensor material with a cotton-based cellulosic filter paper (CFP) and nanocellulose crystals (NC), and these concepts are treated in the context of the functionality of the material properties that target the pathology of the chronic wound [18]. Here the material properties of the sensors are treated from a perspective of characteristic features of compatibility that function at the dressing/wound interface.

## 2. Materials and Methods

### 2.1. General

Sodium chloride (NaCl), potassium chloride (KCl), sodium hydroxide (NaOH), monosodium phosphate (NaH_2_PO_4_) and trifluoroethanol (TFE) was purchased from VWR (Radnor, PA, USA). Promogran Prisma™ (Systagenix, San Antonio, TX, USA) was obtained from HighHealthTide (Santa Rosa, FL, USA). Salt-free lyophilized human neutrophil elastase (HNE) was purchased from Athens Research and Technology (Athens, GA, USA) and the peptide *N*-succinyl-Ala-Pro-Ala-7-amino-4-methylcoumarin (Suc-Ala-Pro-Ala-AMC) from BACHEM (Torrance, CA, USA). Both were used as received without further purification.

### 2.2. Summary of Synthetic Approach of Peptide-Cellulose Conjugates

Peptide immobilization was achieved by reacting the NH2-Glycine-esterified cellulose with the carboxylate of the succinylated-peptide fluorophore (Succinyl-Ala-Pro-Ala-AMC) through a carbodiimide mediated reaction as previously outlined [19]. The resulting product was characterized by removal of a small amount of peptide (Gly-(Succinyl)-Ala-Pro-Ala-AMC) from the peptide-cellulose conjugate materials which was necessary to obtain mass spectroscopic characterization, ([M + H] = 572.374), of the peptide-cellulose conjugate and demonstrate formation of the conjugate bond.

### 2.3. Response and Sensitivity Assay

All of the prepared cellulosic biosensors were subjected to fluorescence response and sensitivity assays as described elsewhere [20]*.* Fluorescence response: In brief, about 2 mg of each biosensor (one repetition) was placed into a cavity of a well plate and 100 μL of phosphate buffer solution (PBS) was added. Further cavities were filled with a dilution series of the tripeptide (1–0.0156 μmol/mL; 100 µL each) next to pure PBS aiming to derive a standard curve. Then, 50 μL human neutrophil elastase (HNE) was added to all samples except for the PBS to trigger the fluorescence response. Sensitivity assay: 50 μL of HNE (2–0.0156 U/mL) was added to another set of dissolution series and biosensors (same amounts as above) to determine the lowest sensitivity detection limit. The fluorescence response of the biosensor samples (λ_exc_ = 360 nm, λ_em_ = 460 nm) was followed for one hour at a resolution of one minute and constant temperature of 37 °C. All samples were shaken for 3 s prior to each measurement. Equipment: Synergy HT, tungsten halogen lamp, photomultiplier detection (BioTek, Winooski, VT, USA). Acquired raw data were processed with the Microsoft Excel 2013 software package (Microsoft Inc., Redmond, WA, USA).

### 2.4. Surface Charge

Surface charge of cellulose filter paper (CFP) was determined by zeta potential (ζ) measurement using Anton Paar Surpass equipment (Ashland, VA, USA). Electrophoretic mobility of CFP was determined in a cylindrical cell. All analyses were conducted using a solution of 1 mM KCl in deionized water (21–23 °C). A 0.1 M NaOH solution was used to adjust the KCl solution to pH 10.3 for CFP. Titration conditions for ζ measurements: 0.100 M HCl, desired pH increment difference 0.200, volume increments 0.020 mL, pH minimum 2.5, pH maximum slightly beyond 10.3, flow rate 50–150 mL/min, P_max_ = 30 kPa. The zeta potential of NC, NA, and the commercially available sequestrant dressing was investigated using a Malvern Zetasizer nano series (Westborough, MA, USA). All data discussed are average values of three independent measurements (PBS, 21–23 °C).

### 2.5. Sequestration of Elastase from Wound-Like Fluid by Cellulosic Transducers

A modified version of the fluorescence response assay described above was used. In brief, 2 mg aliquots of the transducer materials CFP, NC and NA as well as the commercial sequestrant dressing (OSD), (Promogran Prisma™, (Somerville, NJ, USA) were placed in duplicates into cavities of a 96 well plate. Subsequently, a solution of 100 µL HNE (0.5 U/mL) in PBS was added. After 24 h residence time the dressing and transducer materials, respectively, were withdrawn from the respective solutions and squeezed to remove excess of wound-like fluid. The recovered fluid was combined with the fraction that remained in the wells and filled-up to 100 μL using PBS. A dilution series of the tripeptide substrate (1–0.0156 μmol/mL) next to pure PBS was added in 200 µL aliquots to the well plate, too, aiming to derive a standard calibration curve. The reaction was started by adding 200 μL of 0.5 μmol/mL tripeptide substrate solution to the well containing 0.5 U/mL of HNE while 100 μL of the same HNE solution was added to the cavities containing the dilution series of the tripeptide substrate (total volume 300 μL). Monitoring of the fluorescent response was accomplished as described above.

### 2.6. Water Contact Angle (WCA) Measurements

Water contact angle analysis was performed using a video contact angle VCA Optima XE instrument (AST Products, Inc., Billerica, MA, USA). One drop of distilled water was deposited onto the surface of the respective sample. The shape of the droplet was immediately captured and analyzed for the respective contact angle using VCMA optima software. The contact angles discussed in this paper represent average values of three independent measurements at different sites.

### 2.7. Moisture Absorption

Moisture absorption of the transducer materials was studied over a period of 3 h. After submersing in Millipore water, the samples were withdrawn at intervals of 15, 30, 60, 90, 120, and 180 min, slightly dried using a Kimwipe and weighed. The swelling capacity of the transducers was determined as follows:

(1) $$Swelling\;\left(\%\right)=\frac{\left(Ws-Wd\right)}{\left(Wd\right)}*100$$

where Ws = weight of material saturated, Wd = weight of material dry.

### 2.8. Degree of Substitution

The degree of substitution (D.S.) of the cellulose chain is the number of substituent groups attached per anhydroglucose repeating unit (AGU). The D.S. of the biosensors was calculated using Equation (2), where PC is the percentage of nitrogen as determined by elemental analysis, MW_AGU_ is the molecular weight of one cellulose unit, MW_N_ is the molecular weight of one nitrogen atom, N represents the number of nitrogens and MW_PepNA_ is the molecular weight of the peptide including the glycine linker:

D.S. = PC(MW_AGU_)/[(MW_N_)(N)(100) − (MW_PepNA_)] (2)

### 2.9. Specific Surface Area and Average Fibril Diameter

The Brunauer–Emmett–Teller specific surface area (SSA) of NA was determined by nitrogen sorption at 77 K using a Micromeritics ASAP 2405 instrument (Micromeritics, Norcross, GA, USA) [21]. The NA (0.033 g) was degassed in the Micrometrics ASAP 2405 at 100 °C for 4 h prior to the analysis.

The average fibril diameter (*d*) of NA was calculated from the BET specific surface area (Equation (3)) [22], assuming a cylindrical shape of the cellulose fibrils and a skeletal cellulose density (ρ_SK_) of 1.46 g/m^3^:

*d* = 4 (ρ_SK_/S_BET_) (3)

### 2.10. Microscopy Studies of NAs

The morphology of the NA samples was studied by scanning electron microscopy (SEM) using a Tecnai Inspect S50 instrument (FEI, Hillsboro, OR, USA) and an acceleration voltage of 5.00 kV. All images were acquired with a magnification of 1000× (100 μm scale). The NAs were sputter coated with a 6-nm layer of gold using a Leica EM SCD005 instrument (Leica, Buffalo, NY, USA). The Louisiana State University Shared Instrumentation Facility performed the field emission scanning electron microscopy (FE-SEM). The NA was also imaged using an FEI Quanta 3D FEG FIB/SEM instrument (FEI) at a magnification of 65,000× (scale: 500 nm). The NAs was sputter coated with a thin 3-nm layer of gold-palladium using a Leica EM ACE600 instrument (Leica, Buffalo Grove, IL, USA). A thin layer of coating was used to minimize the alteration of the surface morphology.

## 3. Results and Discussion

### 3.1. Structure/Function and Physical Property Considerations

**Figure 1:**
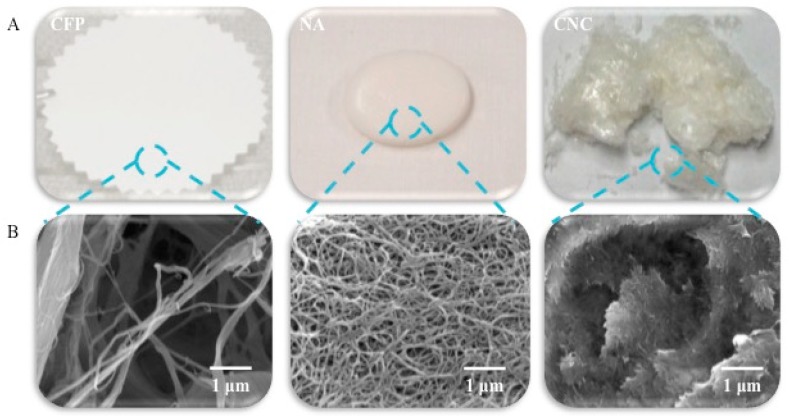
Images of the cellulosic (CFP, blue) and nanocellulosic (NA, pink and NC, green) transducers as (**A**) optical and (**B**) FE-SEM images.

A depiction of the physical and microscopic structure of the materials of this study is shown in Figure 1. The intended application of the nanocellulose aerogel sensor is as an interface with chronic wound dressings. In addition to the cellulose crystallite size as discussed [18], the overall specific surface area, porosity and pore size play a role in sensor sensitivity. Table 1 lists the percent porosity, actual pore size, and specific surface area of the materials of this study. Similarly, in Figure 1 the physical shape and structure of the materials depicted in the FE-SEM images of this study is worthwhile to consider in the context of porosity. In this regard, it is notable that the cellulosic filter paper, which is a relatively planar material and rigid in composition, has a highly porous surface with a moderate porosity (65.6%) and pore size (20–25 μm), however it has a relatively low specific surface area (0.020 m^2^g^−1^). Whereas, the cellulose nanocrystals have a free particle, needle-like shape [23,24] with a higher specific surface area (186.2 m^2^g^−1^) due to the crystalline state of individual monodispersed crystallites. On the other hand, the nanocellulosic aerogel is also relatively planar but has a three dimensional interconnected open porous structure. Thus, the nanocellulosic aerogel has a relatively higher specific surface area (162.9 m^2^g^−1^) than the cellulose filter paper and yet a somewhat lower specific surface area than the cotton nanocellulose crystals. However, the nanocellulosic aerogel’s porosity (99%) is high. Interestingly the nanocellulosic aerogels also have two pore size categories: mesoporous (2–50 nm) and macropores (≥50 nm) [19].

**Figure 2:**
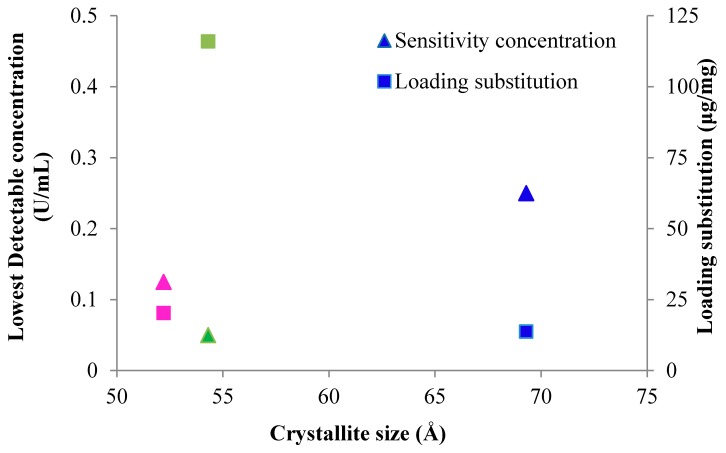
Plot of sensitivity concentration and loading substitution versus the kinetics parameter X-ray diffraction parameter crystallite size. The sensitivity concentration is depicted as triangles and the loading substitution is shown as squares for CFP (blue), NA (pink), and NC (green). Lowest detectable concentrations were CFP (0.250 U/mL), NA (0.125 U/mL), NC (0.050 U/mL). Crystallite size are single point determinations based on X-ray Diffraction Analysis as previously reported [18]. Loading substitution were based on elemental analysis of nitrogen and measured CHN were within 0.40% and run twice to ensure reproducibility as previously reported [19].

The specific surface area of the nanocellulosic materials is inversely related to the crystallite size, and it is clear that the specific surface area (SSA) of the different materials also tends to influence the degree of tripeptide loading on the transducer surfaces, as seen in Figure 2. For instance, the peptide loading to the biosensors (pCFP, pNC, and pNA) is 13.75, 20.31, and 115.94 μg/mg, which directly correlates to the specific surface area values of 0.020, 162.9 and 186.2 m^2^g^−1^, respectively. Thus the specific surface area of the nanocellulose crystals decreases in the order (NC) > nanocellulosic aerogel (NA) > cellulosic material (CFP). Based on the transducer crystallite sizes as discussed in the previous paper, 13%, 14%, and 15% of the total primary hydroxyls are exposed on the crystallite surface of CFP, NA, and NC. So, it follows that the availability of primary hydroxyls on the transducer surfaces also correlates well with the loading of the tripeptide and D.S. levels listed in Table 2. The relationship of specific surface area to D.S. levels in nanocellulosic versus cellulosic sensors as are treated here has been discussed extensively in a previous report where a similar chemistry was employed to apply the peptide substrate to transducer surfaces [25], and it is consistent with the observations of the three transducers discussed here.

#### 3.1.1. Sensor Protease Detection Levels Relevant to Chronic Wounds

The actual sensor sensitivity to levels of detectable elastase in wound fluid reveals the efficacy of the sensors access to protease. Figure 2 relates the relative fluorescent response sensitivity in terms of lowest detectable concentration versus crystallite size and sensor peptide loading. The profile of the three sensors detection sensitivity to elastase from highest to lowest is pNC > pNA > pCFP. The sensitivity of the pCFP, pNA and pNC biosensors expressed as lowest detectable protease concentration was 0.25, 0.13 and 0.05 U/mL, respectively. On the other hand the actual substrate specificity of the enzyme for the tripeptide sequence Ala-Pro-Ala is relatively high based on the COOH-terminal alanine promoting relative enzyme activity 50 percent more than most non-alkyl side chain amino acids, and 30 percent for proline, which is the amino acid favoring relative activity at the P2 site [26]. Moreover the recent report by O’Donoghue [26] also suggests that global substate recognition reveals a consensus motif contributed by elastase, which is the major contributor to neutrophil extracellular traps which form a defense mechanism for neutrophils to invade and ensnare bacteria.

It is interesting that based on previous reports of elastase in chronic wound fluid the nanocellulosic sensor materials are sensitive to HNE at concentrations found in chronic wound fluid [4,5,27]. More importantly, it is clear that the nanocellulose materials (NA and NC) offer improved sensitivity compared to the cellulose filter paper (CFP).

As has previously been observed sensor sensitivity has been correlated with specific surface area when cellulose is contrasted with nanocellulose as a transducer surface [20,25,28]. This type of structure function correlation is apparent here as well. The fluorescence response and sensitivity of the pCFP is consistent with a lower specific surface area (SSA), which also correlates to a lower peptide substitution on the surface of the cellulosic material, Table 2. The SSA of the pNA (163 m^2^g^−1^) and pNC (186 m^2^g^−1^) are comparable but a slightly lower fluorescence response and sensitivity is observed for the pNA. On the other hand, the D.S. level for pNA is fivefold lower than for pNC. Thus, other influences like material swelling and charge as discussed below may play a significant role in sensor sensitivity.

Typically, acute wounds have normal levels of MMPs and HNE [5,29], which facilitates the clearance of cellular debris. However, chronic wounds have elevated levels of both protease and the HNE range (0.02–0.1 U/mL), which depends on the type of chronic wound, i.e., diabetic, venous pressure, and arterial ulcers [16]. Thus, the Serena et al. paper [29] suggests that it is becoming more clinically feasible to track harmful protease levels versus levels that are associated with a trajectory for wound healing. Recently others have reported protease sensor designs for chronic wound applications with protease detection within the range typically expected for chronic wounds [30,31,32]. It is notable that in situ protease sensors may be utilized as theranostic indicators before they become useful for point of care diagnostics in chronic wound care.

It is worth noting that some of these approaches also employ a sensor/dressing design that may be immersed directly in the wound fluid by way of modified hydrogels or optic fibers that are reportedly noninvasive. For example Patrick and Ulijn reported on hydrogel particles with poly (ethylene glycol acrylamide) also employing a peptide sensing element which detects elastase to a concentration of 100 ng/mL, and sequesters the protease effectively removing it from solution [30]. Moreover, Schyrr et al. [31] have recently reported a fiber optic sensor based on the degradation of thin gelatin films which detect matrix metalloproteases at levels of 1–10 micrograms/mL, and also serve as a substrate to lower protease activity. These types of apparent noninvasive designs using modified hydrogels that allow unimpeded aqueous diffusion of the protease into the sensor/dressing matrix have similarities to a cellulosic approach for in situ detection of esterases reported by Derikvand et al. [33]. Alternatively, we have previously proposed an approach utilizing a barrier to prevent uptake of the protease hydrolysis product (free fluorophore or chromophore) into the wound bed [23].

It is also important to note that recent reports on approaches to improve sensor sensitivity by way of increased surface area and biocompatibility on glass, graphene, and biomass-derived nanocomposites have demonstrated the unique utility of techniques, such as nanopatterning, activated carbon transition metal oxides, and graphene nanomesh technology [34,35,36]. These approaches improved biosensor sensitivity above standard protocols for electrodes, receptor-guided biomarkers, and small molecule detection. Thus, it is possible that these innovative approaches to address material surface properties of biosensors may also prove beneficial in protease sensor development. Along these lines the cotton-based aerogel technology demonstrates utility for its improved surface area and high porosity, and as discussed above and below the combination is uniquely suitable for sensor/dressing applications.

#### 3.1.2. Effect of Transducer Porosity and Pore Size on Elastase Detection

**Figure 3:**
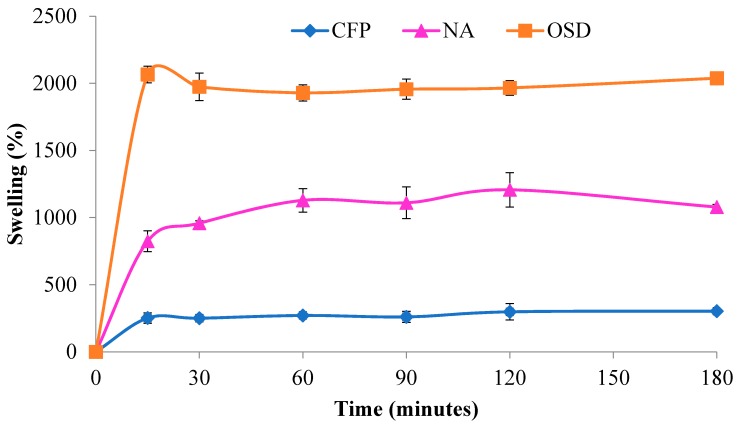
Moisture absorption (Millipore water) of cellulosic filter paper (blue), nanocellulosic aerogel (pink), and commercially available sequestrant dressings (orange).

Material porosity and pore size play a significant role in the functional properties of wound dressing materials as discussed below. However, they are also important from a standpoint of sensor detection based on the relative size of the biomarker protein and its propensity to remain on the surface or penetrate the surface of the transducer. In addition, diffusion of wound exudates into the matrix may bring about swelling resulting in a significant increase in surface area in some materials as shown in Figure 3.

Based on its size the elastase protein is only accessible in theory to the surface of the nanocellulosic aerogel (pNA) but can potentially enter the interior pores of the cellulosic matrix (pCFP). For example, the moderate porosity (65%) and pore size (20–25 μm) of pCFP would readily allow passage of the HNE protein into its interior structure according to its polar surface area value of 309 nm^2^ [20]. This is true in so much as macropores allow passage of the serine protease to the immobilized tripeptide substrate attached to cellulose within the interior structure whereas mesopores would not allow passage of the protease or facilitate reaction with the peptide ligand which has a polar surface area of 2 nm^2^ (note the peptide substrate may be covalently bonded within the nanocellulosic aerogel interior due to its relatively small size) [19]. Thus, access of HNE to the interior of the aerogel may be restricted especially based on considerations of the material’s structure under non-aqueous condition.

**Figure 4:**
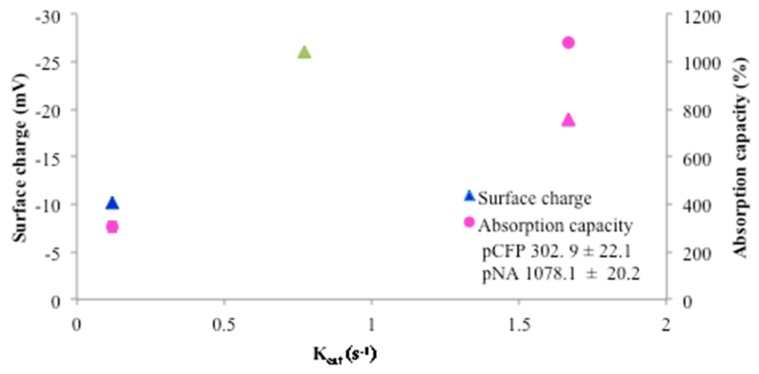
Plot of surface charge and absorption capacity versus the kinetics parameter K_cat_. The surface charge is depicted as triangles and the absorption capacity is shown as circles for CFP (blue), NA (pink), and NC (green). Note actual surface charge values are reported in Table 3. No absorption capacity was determined for NC due to nanocrystalline cellulose composition. k_cat_ (s^−1^) values: NC (0.77), NA (1.67), CFP (0.12).

On the other hand, even though it is not known whether swelling of the nanocellulose aerogel would expand the dimension of the pores to allow entry of the HNE into the nanocellulosic aerogel interior, the swelling as shown in Figure 3 is 1000 percent of the dry weight, and entry of HNE into swollen pore under aqueous conditions is in theory a possibility to some extent. The high porosity and absorption capacity of the aerogel also probably play a role in the improved turnover rate (k_cat_) observed in the previous paper and demonstrated in Figure 4. The k_cat_ value observed is close to the enzyme turnover rate observed in solution. This is consistent with the high porosity and aqueous swelling creating a uniform aqueous environment that approximates that of a single solution phase reaction i.e., higher hydration. In addition as pointed out earlier cellulose II hydrate, which should be present from the aqueous environment of the swollen nanocellulosic aerogel has a larger unit cell than the dehydrated form of cellulose II [37], and may also account to some extent for the increased swelling of the nanocellulosic aerogel. Thus, there may also be some amorphous structure that allows for space to be occupied by water molecules. It is also possible that proteins from wound fluid would compete for internal surface area binding and perhaps become trapped in the nanocellulosic aerogel pores.

Since the pNC is crystalline it does not have a measurable porosity or pore size, and its free particle nature in powder form results in a relatively high SSA providing potential for the greatest degree of surface interaction with HNE, which is consistent with the higher fluorescence response and sensitivity for pNC.

#### 3.1.3. Surface Wetting, Absorption, and Permeability of the Nanocellulosic Aerogel

Chronic wounds are typically poorly hydrated, i.e., ranging from very desiccated to highly exudative. However, since the discovery by Winter in the early sixties [38] that moist wounds heal faster than dry wounds there has been a steady growth in chronic wound dressings [39,40,41] designed to regulate water vapor and gases and promote an optimally moist environment to restore epidermal barrier function. Thus, integral to dressing design is the ability to control moisture evaporation and gas exchange at the wound surface, and sensor function in sync with a compatible design. Dressing and sensor properties that relate directly to wound hydration include wettability, high swelling, water vapor transmission rate, and gas permeability. These characteristic properties are often found in a porous cellulosic material and are central to promoting moist wound healing in the functional design of a semi-occlusive wound dressing.

The interconnected open porous structure of the NA is exemplary of a material that facilitates gas exchange which is also expected to facilitate wound hydration [42]. In this regard, wettability and absorption properties of the NA are relevant to wound fluid absorption. Assessment of the swelling properties of the NA is shown in Figure 3. Imbibition of water by NA leads to a one thousand percent increase in maximum volume over a fifteen minute period and the NA retains its absorption capacity indefinitely, thus demonstrating excellent wicking and moisture retention. It is also compared with other dressing materials that sequester proteases in chronic wounds.

As discussed above the relationship of the materials absorption capacity and charge to the first order rate constants for elastase with the peptide cellulose analogs as a substrate is shown in Figure 4. Here it is also apparent that there is a relation between the higher absorption capacity providing a hydrogel-like or aqueous environment that increases the solvency of the enzyme and peptide binding and thus improves the K_cat_ as reported and discussed [18].

The presence of pectin in the pNA [19] may also promote absorbent properties and it is notable that some commercial hydrocolloid dressings contain gelatin and pectin that contribute to the absorbency [9,19,43]. The contact angle measurements of the CFP and NA were <5°, indicating a high wettability [21], and thus, a highly hydrophilic and absorbent structure. Thus, CFP and NA rapidly wick the aqueous droplet into their cellulosic structure. The high wettability of the NA is due to its cellulosic properties and highly porous structure and is comparable to other cellulosic aerogels reported in the literature [22], which are favorable for the capability to retain wound exudates that may be harmful to wound healing.

The wetting and absorption enhances the absorbent properties of the aerogel transducer surface. Based on the high diffusion rate of the cellulosic aerogels it is expected to have a water vapor transmission rate similar to that of polyacrylamide and polyethylene oxide hydrogels ranging from 9009–9360 g/m^2^/24 h [44,45], which is consistent with high water vapor permeability as it relates to air permeability and a highly porous structure found with the cotton-based aerogel of this study.

#### 3.1.4. Protease Sequestration Activity

The removal of excessive proteolytic activity, which is damaging to growth factors and the extracellular matrix (ECM), in the chronic wound that is essential for completion of the three weeks of healthy wound healing cycle healing [14]. To accomplish removal of proteases from the wound environment a protease sequestrant dressing’s (also referred to as protease-lowering and protease modulating dressing) design may be couched in a number of molecular motifs based on the structural features of the protease. Both the physical properties and molecular design of a dressing can conspire to remove proteases from the wound environment either through unintended or a priori design. The molecular features of the material may be targeted to the protease size, charge, active site, or conformation to enhance selective binding of the protein to the dressing material and to trap and remove proteases from the wound bed [5,13,46,47,48]. Negatively charged cellulosic dressings derivatized by phosphorylation, sulfonation, and carboxylation have previously been shown to increase binding to an arginine-rich and positively charged HNE which is also characteristic of all other neutrophil serine proteases [13,49]. A significant portion of the affinity between the sensor material and HNE is due to charge, and relatively less due to interaction with the peptide portion of the material.

**Figure 5:**
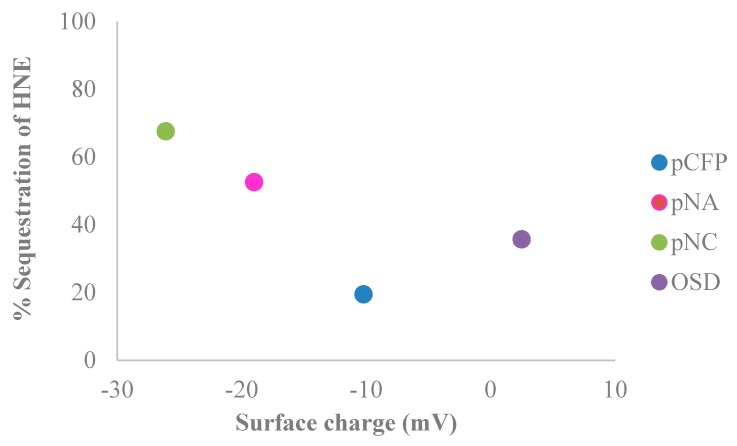
The percentage of sequestered protease removed from 0.5 U/mL of wound-mimicking fluid by the control (black), CFP (blue), NA (pink), NC (green), and a commercially available sequestrant dressing (OSD) (purple). Note 100% sequestration would be complete removal of HNE from solution.

Protease sequestrant activity of the elastase sensors of this study were assessed in conjunction with charge properties determined from electrokinetic analysis (shown in Figure 5 and listed in Table 3), which correlate with the dressing’s ability to remove the positively charged serine proteases as well as matrix metalloproteases from the wound-mimicking solution. The relevant protease sequestrant properties of the nanocellulosic aerogel are contrasted alongside other materials. As shown in Figure 5 and outlined in Table 3 a range of negative charges (−8–−26 mV) correlate with the ability of the material to sequester human neutrophil elastase, a positively charged protein i.e., the ability of the negatively charged material to remove the positively charged HNE from solution. The relative protease sequestrant activity of the sensors evaluated ranged from 20–70% as determined from elastase removed from 0.5 U/mL of wound-mimicking serine protease-containing fluid, i.e., an increased negative charge corresponds with increased removal of protease. However, it is worthwhile to consider that if the elastase can penetrate the interior of a swollen nanocellulosic aerogel material as is indicated in Figure 3 it may also become sequestered to some extent in the aerogel from physical entrapment in the aerogel interior.

Another notable influence on charge in the wound environment is the pH. In this study, the wound-mimicking serine protease containing fluid relates to chronic wound fluid by supplementing phosphate buffered solution with the serine protease at an elevated pH of 7.3. This is important in considering that acute wound fluid typically has a pH of ~4–6 [50] whereas chronic wound fluid has a pH of ~7–8 [51]. Table 3 lists the surface charge of the transducer surface materials including the cellulose filter paper (CFP), nanocellulose crystals (NC), and nanocellulose aerogel (NA) and a commercial protease sequestrant dressing under varying conditions of ionic strength. The zeta potential of the CFP (−10), NA (−19 mV), NC (−26 mV), and protease sequestrant dressing (3 mV) at low ionic strength (DI-H_2_O, 3.51 μS/cm) is reflected in a greater negative surface charge conferred to the nanocellulosic materials (NA and NC) than the cellulose and oxidized cellulose/collagen materials (CFP and protease sequestrant dressing).

#### 3.1.5. Biosensor Properties as a Function of an Intelligent Semiocclusive Dressing Motif

The cellulosic and nanocellulosic biosensors (pCFP, pNA and pNC) have different structural features and detection sensitivities as shown in Figure 1 and Figure 2. In terms of the more planar but porous pCFP its detection sensitivity (0.25 U/mL) is above the threshold of detecting HNE at concentrations present in chronic wound fluid (0.02–0.1 U/mL), however it may be considered useful as a detector for highly concentrated proteases to signal time to change in a protease dressing motif.

The free particle crystalline nature of the pNC has a high sensitivity. This system may be more beneficial in a dip-stick or swab design versus a dressing design [23]. On the other hand, the three dimensional NA structure is compatible for a biosensor layer and has a detection sensitivity (0.13 U/mL) capable of detecting HNE in wound fluid. Therefore, the pNA biosensor may function as a component of a multilayered protease sequestrant dressing.

**Figure 6:**
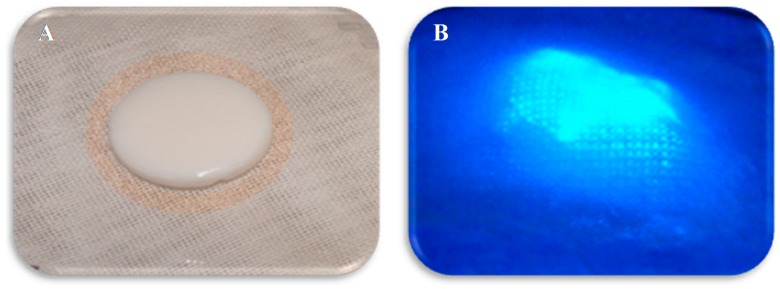
Depiction of pNA conjugate biosensor with protease activation that is interfaced in a multilayer bandage prototype both with and without ultraviolet activation [52]. Image (**A**,**B**): demonstrates the gauze contact layer and the aerogel biosensor layer with and without ultraviolet.

Figure 6 shows a layered pNA dressing with semiocclusive properties depicted as it might appear when detecting dressing saturation with proteases from a chronic wound. The multilayered dressing consists of a contact layer of gauze, a biosensor layer in between two barrier layers, and a top layer. The wound contact layer also functions by design as a protease sequestrant. The contacting layer absorbs wound exudate and is accompanied by a dialysis membrane as previously described [23], which acts as a dialyzer of the elastase protease from wound fluid; the pNA conjugate biosensor layer activates at the sensor threshold of proteases (0.13 U/mL), sequesters the HNE protein present in the wound fluid, and releases the AMC fluorophore when in contact with HNE. The barrier layer is positively charged and binds the released fluorophore from entering into the wound bed. The top layer acts as an overlay to protect the sensor dressing and wound bed. This type of prototype dressing might either be employed to signal time to change the dressing or over time monitor the likely progress of wound healing as measured by protease secretion. Thus, multilayered sensor-based protease sequestrant dressings of this type would provide an optimal approach to combine characteristic properties of wound dressings while targeting harmful proteases present in chronic wounds.

### 3.2. Future Prospects

As portrayed here the protease sensor/sequestrant technology has potential to be useful as a POC detection of HNE activity. However, optimization of the protease recognition consensus sequence of the sensor peptide would improve cross-detection of other neutrophil proteases like MMPs. As shown previously by applying this same approach to colorimetric POC [23] protease detection a visual color assessment would eliminate the need for a UV source to visualize signaling of the presence of proteases at POC. A colorimetric approach to wound protease levels would also allow detection of protease titer present in the dressing through inhibitor doping of the sensor transducer surface to create localized points on the material by adjusting contrasts of sensitivity with degrees of color. This approach would be useful in discerning how saturated the dressing is and the degree of wound infiltration of proteolytic activity. It is important to note that future efforts to translate these types of technology into the clinic face challenges in selecting patient populations where a broad range of chronic wounds can be assessed for the types of sensor applications that clinicians and researchers can sort through to make the technology practical in guiding treatment and predicting outcomes [53].

## 4. Conclusions

We have evaluated cellulosic (CFP) and nanocellulosic (NA and NC) materials as transducer surfaces for biosensors that are able to detect and sequester proteases as a dressing interface for chronic wounds. The materials (cellulosic filter paper, nanocellulosic aerogels, and cellulose nanocrystals) were selected because of: (i) their structural properties; (ii) their favorable permeability, moisture retention, and charge properties; and (iii) their SSA and surface charge for sequestration purposes. Characterization of the CFP, NA, and NC transducers indicated porous structures with high porosities and varying pore sizes, and high SSA structure. The wetting and absorbent properties of the transducers are necessary for uptake of wound exudate. Furthermore, the morphology of the cotton based CFP and NC are dominated by cellulose I diffraction patterns whereas the cotton derived NA is dominated by the fibrous cellulose II network.

Conjugation of the materials to a tripeptide substrate provided the selectivity and sensitivity needed for detecting HNE at concentrations present in chronic wound fluid in particular, for the pNA and pNC. Both of the nanocellulosic materials (pNA and pNC) had higher levels of peptide loading, surface charge, and protease sequestration than CFP. Therefore, nanocellulosic materials are promising transducer surfaces for biosensors. The higher SSA and surface charge parallel the peptide loading and protease sequestration, which alters the fluorescence response and detection sensitivity. The nanocellulosic pNA and NC biosensors detect HNE at concentrations found in chronic wounds. Although pNA has a limited sensitivity concentration of 0.13 U/mL and is able to detect HNE at levels found in arterial chronic wound fluid (0.1 U/mL), expanding its sensitivity concentration (<0.1 U/mL) to target all chronic wound types (diabetic, venous, and pressure wounds) would be ideal.

## Figures and Tables

**Table 1:** Porosity, average pore diameter, and specific surface area (SSA) values obtained for the CFP, NA, and NC.

Name	Porosity ^a^	Average Pore Diameter	SSA ^c^
	(%)	(nm)	(m^2^g^−1^)
CFP	65.6	20–25 ^b^	0.020
NA	98.8	11	162.943
NC	-	-	186.200

a: The porosity and pore diameter of the CFP is according to its properties listed on Sigma Aldrich website whereas the porosity and average pore diameter of the NA were calculated using nitrogen adsorption with the Brunauer-Emmet-Teller theory. b: The average pore diameter is in μm. c: The specific surface area (SSA) for the Whatman filter paper (CFP) and NC are based on the number of particles and specific surface area of cylinder formula. The SSA of the NA was calculated using nitrogen adsorption with the Brunauer-Emmet-Teller theory.

**Table 2:** Degree of substitution (DS) of the cellulose and nanocellulose sensors.

Name ^a^	D.S. ^b^
pCFP	0.014
pNA	0.015
pNC	0.075

a: p = *N*-succinyl-Ala-Pro-Ala-7-amino-4-methylcoumarin, CFP = cellulosic filter paper, NA = nancellulosic aerogel, NC = nanocrystalline cellulose. b: Degree of substitution of peptide.

**Table 3:** Surface charge and percent of sequestration of the cellulose, nanocellulose, and the commercially available OSD.

Name	Surface Charge	Sequestration of HNE
	(mV)	(%)
pCFP	−10.2 ± 0.6	20 ± 1.0
pNA	−19.0 ± 0.6	53 ± 1.0
pNC	−26.1 ± 0.1	68 ± 4.0
OSD	2.5 ± 0.1	36 ± 3.0
